# Risk factors for mechanical complications in very elderly patients with acute myocardial infarction

**DOI:** 10.3389/fmed.2025.1714080

**Published:** 2025-12-02

**Authors:** Luqin Yan, Wei Yuan

**Affiliations:** 1 Department of Cardiovascular Surgery, The First Affiliated Hospital of Xi’an Jiaotong University, Xi’an, China; 2 Department of Cardiovascular Medicine, The First Affiliated Hospital of Xi'an Jiaotong University, Xi’an, China

**Keywords:** acute myocardial infarction, mechanical complications, very elderly, risk factors, prognosis

## Abstract

**Background:**

The mortality rate for mechanical complications in very elderly acute myocardial infarction (AMI) patients is extremely high, but predictive tools specifically for this population are lacking.

**Methods:**

Mechanical complications (free-wall rupture, ventricular septal rupture, papillary muscle rupture/dysfunction, ventricular aneurysm) were independently validated. Differences in baseline characteristics, laboratory markers, and outcomes were compared. Missing data underwent sensitivity analysis, with >20% exclusion and <15% imputation. Regression modeling entered variables with univariate association (*p* < 0.05) or clinical relevance into multivariable logistic regression; final model derived via backward elimination (*p* < 0.05 retained) with VIF > 5 exclusion. Analyses used SPSS/GraphPad (two-tailed *p* < 0.05).

**Results:**

This retrospective cohort study analyzed 2,467 consecutive AMI patients aged ≥ 75 years. Mechanical complications occurred in 9.6% (*n* = 236) of patients. Ventricular aneurysm (VA) predominated (92.8%), strongly associated with anterior infarcts (71.2%, *p* < 0.001) and reperfusion (81.7%, *p* < 0.001). Rupture complications favored non-reperfused infarcts. Patients with mechanical complications exhibited distinct profiles: lower systolic blood pressure (115.9 vs. 123.5 mmHg, *p* = 0.001), higher STEMI prevalence (60.6% vs. 44.1%, *p* < 0.001), advanced Killip class III-IV (22.5% vs. 13.3%, *p* < 0.001), and biomarker evidence of intense inflammation (elevated WBC, neutrophil%, hs-CRP), myocardial injury (higher cTnT, NT-proBNP), and metabolic derangements (hypoalbuminemia, hyperkalemia). Multivariable analysis identified independent predictors: Killip class III/IV (OR = 2.99), elevated neutrophil percentage (OR = 1.05), hyperkalemia (OR = 1.70), and hypoalbuminemia (OR = 0.92). A history of hypertension was paradoxically protective (OR = 0.50).

**Conclusion:**

This study identifies ventricular aneurysm as the dominant mechanical complication in very elderly AMI patients, establishes a paradoxical protective role of hypertension history, and proposes a distinct risk profile integrating hemodynamic status, neutrophilic inflammation, and metabolic derangement. These findings are hypothesis-generating and highlight a potentially valuable stratification tool that warrants prospective validation in external cohorts before clinical application.

## Introduction

Mechanical complications of acute myocardial infarction (AMI)—encompassing free wall rupture (FWR), ventricular septal rupture (VSR), ventricular aneurysm (VA), and papillary muscle rupture (PMR)—represent catastrophic syndromes with >90% mortality without emergent intervention (1). The global aging epidemic has amplified this threat, as adults ≥75 years now constitute the fastest-growing AMI subgroup. In this frail cohort, MC incidence exceeds 9% (3 × higher than younger patients) (2, 3), with mortality persistently exceeding 60% despite modern therapies (1). This vulnerability reflects an intersection of age-related myocardial fragility (4), multimorbidity burden (5), and frequent exclusion from guideline-directed interventions due to bleeding risks or functional limitations—creating a critical care paradox where those at highest risk receive least aggressive prevention (6).

Current risk stratification remains dangerously inadequate. Tools like the GRACE score, designed for general AMI mortality, overlook MC-specific pathways by omitting geriatric physiology markers (e.g., inflammatory/oxidative stress biomarkers), underweighting anterior infarction topography, which accounts for 80% of ruptures, and ignoring dynamic tissue instability indicators (7–9). While fragmented evidence identifies isolated risks—female sex, delayed reperfusion, renal impairment—no system integrates these with geriatric-specific vulnerabilities to forecast Mechanical complications (10, 11). This gap perpetuates reactive rather than preemptive care: over 40% of elderly MC diagnoses occur post-cardiac arrest.

The therapeutic window for MC survival is measured in hours, demanding pre-symptomatic recognition (12). Surgical repair remains the only definitive therapy, yet delayed referral contributes to >75% perioperative mortality in the very elederly (13). Given that each 6-h delay doubles mortality risk, we propose developing the first dedicated MC risk score for the very elderly (14). This instrument will synthesize geriatric clinical profiles, infarct characteristics, and emerging biomarker panels to stratify real-time rupture probability. Its implementation could redirect care pathways through protocol-driven escalation of surveillance, expedited revascularization thresholds, and direct bridges to surgical centers—addressing an unmet need in precision geriatric cardiology.

## Methods

### Study population

**Figure 1:**
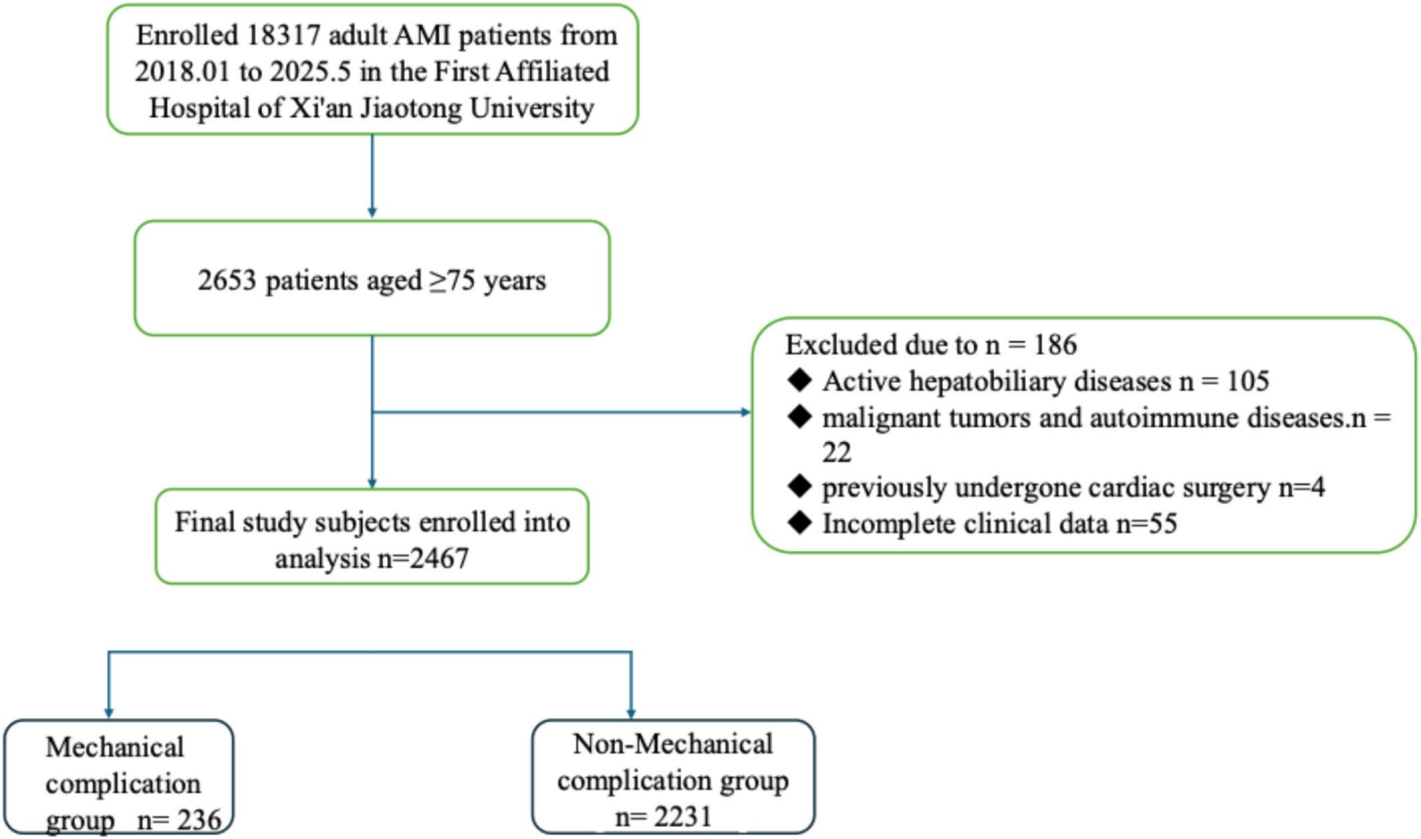
Flowchart of the enrolled patients.

This retrospective cohort study screened consecutive patients aged ≥75 years who were admitted to the First Affiliated Hospital of Xi’an Jiaotong University with a first-time diagnosis of AMI between January 2018 and June 2025 Patients were included according to the following criteria: (1) age ≥75 years at presentation; and (2) confirmed diagnosis of AMI based on the Fourth Universal Definition of Myocardial Infarction jointly issued by the European Society of Cardiology, American College of Cardiology, American Heart Association, and World Heart Federation in 2018. Key exclusion criteria comprised: (1) Mechanical complications attributable to non-ischemic causes (e.g., traumatic cardiac rupture); (2) pre-admission occurrence of mechanical complications (*n* = 23) (Supplementary Table 1); (3) anticipated survival < 24 h due to non-cardiac conditions (e.g., terminal malignancy); and (4) incomplete clinical documentation. To provide full transparency, we have explicitly stated that the reported incidence of 9.6% reflects the rate of in-hospital development of complications. After applying the exclusion criteria, a total of 2,467 patients constituted the final study cohort for analysis (Figure 1).

The study protocol received approval from the Ethics Committee of the First Affiliated Hospital of Xi’an Jiaotong University on 22 July 2025. (No. XJTU1AF2025LSYY-619).

### Definitions and grouping

The final analytical cohort was stratified into two groups based on the in-hospital development of mechanical complications: the complication group (patients experiencing complications) and the non-complication group (patients without complications).

The diagnosis of in-hospital mechanical complications of AMI was rigorously adjudicated according to established clinical, echocardiographic, and/or surgical criteria. These complications encompassed: (1) cardiac FWR, defined as echocardiographic or surgical evidence of myocardial tear with pericardial effusion leading to tamponade; (2) VSR, confirmed by color Doppler demonstration of left-to-right shunting across the interventricular septum; (3) papillary muscle rupture or dysfunction, diagnosed by acute severe mitral regurgitation with direct visualization of rupture on echocardiography or at surgery, or flail leaflet with ruptured chordae; and (4) VA formation, identified as persistent diastolic dyskinesia or akinetic segment with distinct geometric distortion of the left ventricular contour on imaging.

### Statistical analysis

In our analysis, continuous variables were assessed for normality using the Kolmogorov–Smirnov test. Normally distributed data are presented as mean ± standard deviation and analyzed by independent samples t-test, while non-normally distributed variables are expressed as median (interquartile range, IQR) and compared using the Mann–Whitney U test for two-group comparisons. Categorical variables were handled as follows: ordered categories employed the Mann–Whitney U test, whereas nominal variables were reported as frequencies (percentages) and analyzed using Pearson’s χ^2^ test or Fisher’s exact test for sparse data (expected cell counts <5). Regarding missing data, a systematic evaluation revealed a spectrum of missingness across variables. Consistent with our pre-defined protocol, variables with substantial missingness exceeding 20%—including anthropometric measures such as height and weight—were deemed to have insufficient data for reliable analysis and were therefore excluded *a priori* from all univariate screening and subsequent multivariate modeling. This conservative threshold was implemented to prevent potential bias and instability from imputing large portions of data. For variables with missingness below 20%, we implemented a tiered strategy. Killip class, a key clinical severity indicator, had a missingness rate of 18.2%; although below the exclusion threshold, its near-20% missingness warranted careful handling via multiple imputation to preserve statistical power and reduce potential selection bias. Other critical laboratory and hemodynamic parameters—including albumin (ALB), hemoglobin (HGB), fasting blood glucose (GLU), and systolic blood pressure (SBP)—exhibited minimal missingness (each below 5%), making them highly suitable for imputation. To address missing values in these retained variables, we employed Fully Conditional Specification, also known as Multiple Imputation by Chained Equations (MICE), a flexible and widely recognized approach for multivariate missing data in clinical research. The process involved creating 20 imputed datasets with 50 iterations per imputation to ensure convergence, verified by visual inspection of trace plots. The imputation model included all variables intended for the final multivariate logistic regression analysis, including the outcome variable (occurrence of any mechanical complication), to preserve underlying relationships. Appropriate univariate imputation models were specified for each variable type: Predictive Mean Matching (PMM) for continuous variables and logistic regression for binary variables. After separate analysis of each imputed dataset, final parameter estimates were obtained by pooling results using Rubin’s rules. To assess robustness, we conducted a sensitivity analysis comparing the pooled results from the imputed data with a complete-case analysis, which yielded qualitatively similar conclusions. For regression modeling, all clinically plausible candidate variables (demographic, clinical, and laboratory factors) underwent univariate logistic regression to report crude odds ratios (ORs) with 95% confidence intervals (CIs). Variables showing univariate association (*p* < 0.05) or established clinical relevance were entered into multivariable logistic regression. In direct response to reviewer concerns, we have provided a clear rationale for employing stepwise backward elimination (retention: *p* < 0.05), deliberately chosen to develop a parsimonious and clinically interpretable model while mitigating overfitting, given the ratio of candidate predictors to the number of observed endpoint events (*n* = 215). Multicollinearity was assessed by variance inflation factors (VIF > 5 triggering exclusion). Furthermore, acting on the reviewer’s recommendation, we conducted a comprehensive sensitivity analysis by constructing a full multivariate model containing all pre-specified clinical covariates. The comparison convincingly demonstrated that the five key independent predictors in our final model exhibited highly consistent effect estimates and maintained strong statistical significance in the full model, robustly validating the stability of our core findings. Additionally, the superior performance of our final model was objectively indicated by its lower Akaike and Bayesian Information Criterion values, confirming its optimal balance of fit and parsimony. Mechanical complications were analyzed primarily as a composite endpoint (any MC vs. no MC) due to sample size considerations. Given the predominance of ventricular aneurysm and low event counts for other individual complications (free-wall rupture, ventricular septal rupture, and papillary muscle rupture), formal comparative subgroup analyses between specific MC types were not statistically feasible and were not performed; any presentations regarding non-aneurysm complications are purely descriptive and exploratory. All analyses were performed using SPSS 26.0 (IBM, USA) and GraphPad Prism 9.0 (GraphPad Software, USA), with statistical significance defined as two-tailed *p* < 0.05.

## Results

### Baseline characteristics and clinical outcomes

A cohort of 2,467 very elderly AMI patients were enrolled, comprising 1,621 males (65.7%) and 846 females (34.3%). A total of 236 (9.6%) patients developed mechanical complications, with VA overwhelmingly predominant (92.8%, *n* = 213), followed by PMR (3.4%, *n* = 8), VSR (3.4%, *n* = 8), and FWR (3.0%, *n* = 7). Distinct predictor patterns emerged across complication subtypes. VSR demonstrated pronounced association with anterior infarct location (75.0% of cases) and absence of reperfusion therapy (50.0% in non-reperfused patients), while PMR occurred exclusively in non-anterior territories (100%, *p* = 0.002 vs. anterior). Crucially, among all 236 very elderly AMI patients with mechanical complications, the distribution of mechanical complications was significantly associated with baseline renal function defined by an estimated glomerular filtration rate (eGFR) threshold of 45 mL/min/1.73m^2^ (*p* < 0.001). The vast majority of patients (91.5%, *n* = 216) had reduced renal function (eGFR ≤45 mL/min/1.73m^2^). Among patients with mechanical complications, those with reduced renal function accounted for 75.0% of VSR cases, 100.0% of FWR cases, and 87.5% of PMR cases. Conversely, the minority of patients with preserved renal function (8.5%, *n* = 20) constituted 25.0% of VSR cases and 12.5% of PMR cases, with no FWR cases (0%) occurring in this group. Therefore, patients with reduced renal function formed the predominant population across all types of complications (Table 1).

**Table 1:** Distribution of mechanical complications in very elderly AMI patients.

Variable	Overall (*n =* 236)	VSR (*n =* 8)	FWR (*n* = 7)	PMR (*n* = 8)	VA (*n* = 213)
Infarct location					
Anterior MI	162 (68.6%)	6 (75.0%)	4 (57.1%)	0	153 (72.0%)
Non-anterior MI	74 (31.4%)	2 (25.0%)	3 (42.9%)	8 (100%)	60 (28.2%)
Reperfusion status					
Reperfusion therapy	191 (80.9%)	4 (50.0%)	4 (57.1%)	7 (87.5%)	172 (80.8%)
No reperfusion therapy	45 (19.2%)	4 (50.0%)	3 (42.9%)	1 (12.5%)	41 (19.2%)
Renal function					
eGFR < 45 mL/min/1.73m^2^	216 (91.5%)	6 (75.0%)	7 (100.0%)	7 (87.5%)	112 (52.6%)
eGFR ≥ 45 mL/min/1.73m^2^	20 (8.5%)	2 (25.0%)	0	1 (12.5%)	101 (47.4%)

AMI, acute myocardial infarction; CKD, chronic kidney disease; eGFR, estimated glomerular filtration rate; MI, myocardial infarction, VA, ventricular aneurysm, VSR, ventricular septal rupture, PMR, papillary muscle rupture, FWR, free wall rupture.

We next investigated whether there were any significant sex-based differences in the incidence and pattern of mechanical complications. Among the 2,467 patients in the final cohort, the overall incidence of in-hospital mechanical complications was numerically higher in female patients (10.9%, 92/846) compared to male patients (8.9%, 144/1621), though this difference was not statistically significant (*p* = 0.085). However, a striking and statistically significant disparity was observed in the type of complications. Female patients suffered a markedly higher incidence of cardiac rupture (0.59% vs. 0.12% in males, *p* = 0.024), while the rates of other complications, including ventricular septal rupture, papillary muscle rupture, and left ventricular aneurysm, were comparable between the two sexes (Supplementary Table 2).

Stratification of the study population by age revealed a significant graded increase in the risk of mechanical complications, with incidences of 6.86% (713/10391) for patients under 65 years, 8.66% (408/4710) for those aged 65 to 75 years, and 9.57% (236/2467) for those over 75 years. The profile of complications also varied with age; left ventricular aneurysm was the overwhelmingly predominant type across all strata, constituting over 89% of complications in each group, while the proportional representation of other complications showed slight variations (Supplementary Table 3).

We conducted a comprehensive analysis of the complication spectrum in 2467 elderly patients aged over 75 years with acute myocardial infarction (AMI). While structural complications (9.57%) were predominantly left ventricular aneurysms with rare acute ruptures, functional complications were more prevalent (16.01%). These included severe left ventricular dysfunction (12.00%), new-onset atrial fibrillation/flutter (9.00%), alongside considerable rates of right ventricular dysfunction (4.99%), sustained ventricular tachycardia/fibrillation (4.01%), and high-grade AV block (4.01%) (Supplementary Table 4).

Among the 2,467 very elderly AMI patients, significant differences emerged between the MC group (*n* = 236) and the non-MC group (*n* = 2,231). Demographically, groups were comparable in age (79.77 ± 3.96 vs. 79.44 ± 4.06 years, *p* = 0.218) and sex distribution (61.0% vs. 66.2% male, *p* = 0.110). However, patients developing mechanical complications had a significantly lower prevalence of hypertension history (53.0% vs. 63.0%, *p* = 0.002), while histories of T2DM, stroke, and kidney disease showed no significant intergroup differences. Clinically, the complication group exhibited higher admission heart rates (81.1 ± 16.6 vs. 75.7 ± 15.2 /min, *p* = 0.234) and significantly lower average systolic blood pressure (115.90 ± 20.07 vs. 123.45 ± 21.58 mmHg, *p* = 0.001), though diastolic pressure and echocardiographic parameters (LVEF, LVESD, LVSDD) were comparable. Infarction characteristics revealed profound disparities: STEMI presentation was markedly more frequent in the complication group (60.6% vs. 44.1%, *p* < 0.001), and these patients had substantially higher Killip class severity (Class I: 34.7% vs. 59.2%; Class III-IV: 22.5% vs. 13.3%, *p* < 0.001). No significant differences were observed in the number of diseased vessels, primary treatment modality, or hospital length of stay (5.16 ± 3.68 vs. 5.15 ± 4.06 days, *p* = 0.986). A history of dysrhythmia was present in 440 patients (17.8%) and pre-existing heart disease in 231 patients (9.4%). These conditions were more prevalent in patients who developed mechanical complications (dysrhythmia: 33.5%, 79/236; heart disease: 24.1%, 57/236) compared to those without complications (dysrhythmia: 16.2%, 361/2231; heart disease: 7.8%, 174/2231) (Table 2).

**Table 2:** Baseline characteristics and clinical outcomes.

Variable	Overall (*n* = 2,467)	Mechanical complications group (*n* = 236)	Non-complications group (*n* = 2,231)	*p*
Demographics				
Age, years	79.47 ± 3.97	79.77 ± 3.96	79.44 ± 4.06	0.218
Male, *n* (%)	1,621 (65.7%)	144 (61.0%)	1,477 (66.2%)	0.110
Clinical history				
History of HTN, *n* (%)	1,531 (62.1%)	125 (53.0%)	1,406 (63.0%)	0.002
History of T2DM, *n* (%)	812 (32.9%)	69 (29.2%)	743 (33.3%)	0.206
History of stroke, *n* (%)	387 (15.7%)	32 (13.6%)	355 (15.9%)	0.345
History of kidney disease, *n* (%)	119 (4.8%)	12 (5.1%)	107 (4.8%)	0.844
History of Dysrhythmia	440 (17.8%)	79 (33.5%)	361 (16.2%)	0.523
Pre-existing heart disease	231 (9.4%)	57 (24.1%)	174 (7.8%)	0.479
Cardiovascular parameter				
HR, /min	76.2 ± 15.4	81.1 ± 16.6	75.7 ± 15.2	0.234
Avg SBp	122.74 ± 21.55	115.90 ± 20.07	123.45 ± 21.58	0.001
Avg DBp	70.69 ± 12.51	79.13 ± 13.48	76.29 ± 14.15	< 0.001
LVEF (%)	50.2 ± 10.8	50.8 ± 11.5	49.8 ± 11.4	0.218
LVESD (mm)	38.1 ± 7.2	38.1 ± 7.3	38.2 ± 7.4	0.366
LVSDD (mm)	51.6 ± 7.0	51.9 ± 7.7	51.4 ± 6.8	0.440
Infarction characteristics				
STEMI, *n* (%)	1,126 (45.6%)	143 (60.6%)	983 (44.1%)	< 0.001
Killip Class				< 0.001
I	1,403 (56.9%)	82 (34.7%)	1,321 (59.2%)	
II	714 (28.9%)	101 (42.8%)	613 (27.5%)	
III-IV	350 (14.2%)	53 (22.5%)	297 (13.3%)	0.002
Lesion vessels				0.572
1	193 (7.8%)	19 (8.1%)	174 (7.8%)	
2	455 (18.4%)	40 (16.9%)	415 (18.6%)	
3	1819 (73.7%)	177 (75.0%)	1,642 (73.6%)	
Treatment				0.427
Medicine	42 (1.7%)	4 (1.7%)	49 (2.2%)	
PCI/PTCA	2,373 (96.2%)	229 (97.0%)	2082 (93.3%)	
CABG	52 (2.1%)	3 (1.3%)	100 (4.5%)	
Hospital stays	5.15 ± 4.03	5.16 ± 3.68	5.15 ± 4.06	0.986

Avg SBp, Average Systolic Blood Pressure; Avg DBp, Average Diastolic Blood Pressure, HTN, Hypertension; T2DM, Type 2 Diabetes Mellitus.

### Laboratory parameters and biomarker profiles

Table 3 presents the comprehensive laboratory and biomarker analyses, demonstrating significant differences between patients with mechanical complications and those without (Table 3). The complication group exhibited pronounced activation of inflammatory pathways, evidenced by significantly higher white blood cell count (WBC: 8.80 ± 4.07 vs. 8.21 ± 3.61 × 10^9^/L, *p* = 0.019), neutrophil percentage (NEU%: 76.86 ± 10.00 vs. 75.16 ± 10.48, *p* = 0.016), and high-sensitivity C-reactive protein (hs-CRP: 5.94 ± 3.63 vs. 5.23 ± 3.59 mg/L, *p* = 0.006). Markers of myocardial injury and stress were substantially elevated: cardiac troponin T (cTnT median: 0.41 vs. 0.29 ng/mL, *p* < 0.001) and N-terminal pro-B-type natriuretic peptide (NT-proBNP median: 3992.00 vs. 1308.00 pg/mL, *p* < 0.001) were significantly higher in the complication group. A prothrombotic state was indicated by elevated fibrinogen (FIB: 4.12 ± 1.28 vs. 3.79 ± 1.21 g/L, *p* < 0.001) and D-dimer (median: 1.08 vs. 0.76 mg/L, *p* = 0.046). Notable metabolic derangements included lower serum calcium (2.15 ± 0.15 vs. 2.20 ± 0.17 mmol/L, *p* = 0.026), higher potassium (4.15 ± 0.51 vs. 4.01 ± 0.49 mmol/L, *p* = 0.028), reduced albumin (ALB: 35.83 ± 5.91 vs. 36.80 ± 5.03 g/L, *p* = 0.005), and lower LDL-C (2.04 ± 0.88 vs. 2.16 ± 0.84 mmol/L, *p* < 0.001). Arterial blood gas analysis revealed lower PCO₂ (33.39 ± 6.65 vs. 36.63 ± 5.39 mmHg, *p* < 0.001) and higher pH (7.42 ± 0.06 vs. 7.40 ± 0.05, *p* = 0.011) in the complication group. Lipoprotein(a) was also elevated (344.94 ± 268.16 vs. 313.07 ± 272.70 g/L, *p* = 0.018).

**Table 3:** Laboratory parameters and biomarker profiles.

Variable	Overall (*n* = 2,467)	Mechanical complications group (*n* = 236)	Non-complications group (*n* = 2,231)	*p*
HGB, g/L	126.46 ± 19.69	126.53 ± 19.23	126.45 ± 19.74	0.957
WBC, ×10^9^/L	8.26 ± 3.66	8.80 ± 4.07	8.21 ± 3.61	0.019
NEU%	75.33 ± 10.44	76.86 ± 10.00	75.16 ± 10.48	0.016
AB, mmol/L	22.63 ± 3.38	21.72 ± 4.65	22.69 ± 3.28	0.037
pH	7.40 ± 0.05	7.42 ± 0.06	7.40 ± 0.05	0.011
SpO_2_, %	95.02 ± 5.89	95.05 ± 7.54	95.01 ± 5.78	0.960
PO_2_, mmHg	87.12 ± 27.55	90.27 ± 28.53	86.92 ± 27.50	0.377
PCO_2_, mmHg	36.44 ± 5.52	33.39 ± 6.65	36.63 ± 5.39	< 0.001
LDL-C, mmol/L	2.15 ± 0.84	2.04 ± 0.88	2.16 ± 0.84	< 0.001
HDL-C, mmol/L	0.99 ± 0.25	0.99 ± 0.28	0.99 ± 0.25	0.923
lpA, g/L	316.24 ± 272.36	344.94 ± 268.16	313.07 ± 272.70	0.018
Glu, mmol/L	8.08 ± 3.71	8.08 ± 3.69	8.40 ± 3.91	0.163
HbA1c, %	6.62 ± 1.38	6.63 ± 1.40	6.54 ± 1.18	0.333
cTnT, ng/mL	0.31 (0.06, 1.09)	0.41 (0.09, 1.68)	0.29 (0.06, 1.05)	< 0.001
NT-proBNP, pg/mL	1643.00 (560.30, 4140.90)	3992.00 (1577.25, 7298.25)	1308.00 (462.88, 3570.25)	< 0.001
CK-MB, U/L	22.10 (13.00, 59.00)	20.68 (12.55, 49.82)	21.00 (12.60, 52.22)	0.369
CK, U/L	173.00 (80.00, 559.50)	139.00 (70.50, 483.00)	152.00 (77.00, 443.25)	0.915
LDH, U/L	274.00 (2169.00, 407.0)	295.50 (227.25, 467.50)	266.00 (217.00, 366.25)	0.005
AST, U/L	38.00 (24.00, 83.00)	34.00 (23.00, 75.75)	36.00 (24.00, 70.00)	0.319
ALT, U/L	26.00 (18.00, 39.00)	25.00 (18.00, 37.75)	26.00 (18.00, 38.00)	0.616
BUN, mmol/L	7.22 ± 3.80	7.40 ± 3.34	7.20 ± 3.85	0.443
CRE, μmol/L	73.00 (59.00, 94.00)	78.00 (60.00, 93.75)	72.00 (58.00, 90.00)	0.021
ALB, g/L	36.71 ± 5.02	35.83 ± 5.91	36.80 ± 5.03	0.005
Mg^2+^, mmol/L	1.00 ± 0.13	0.99 ± 0.13	1.00 ± 0.13	0.730
Ca^2+^, mmol/L	2.20 ± 0.17	2.15 ± 0.15	2.20 ± 0.17	0.026
K^+^, mmol/L	4.02 ± 0.50	4.15 ± 0.51	4.01 ± 0.49	0.028
P^−^, mmol/L	0.95 ± 0.28	0.98 ± 0.35	0.95 ± 0.28	0.451
FIB, g/L	3.82 ± 1.22	4.12 ± 1.28	3.79 ± 1.21	< 0.001
D-D, mg/L	0.80 (0.53, 1.44)	1.08 (0.70, 2.30)	0.76 (0.51, 1.30)	0.046
hs-CRP, mg/L	5.29 ± 3.60	5.94 ± 3.63	5.23 ± 3.59	0.006

HGB, Hemoglobin; WBC, White Blood Cell Count; NEU%, Neutrophil Percentage; AB, Actual Bicarbonate; pH, Potential of Hydrogen; SpO₂, Peripheral Oxygen Saturation; PO₂, Partial Pressure of Oxygen; PCO₂, Partial Pressure of Carbon Dioxide; LDL-C, Low-Density Lipoprotein Cholesterol; HDL-C, High-Density Lipoprotein Cholesterol; Lp (a) – Lipoprotein (a); Glu, Glucose; HbA1c, Glycated Hemoglobin; cTnT, Cardiac Troponin T; NT-proBNP, N-terminal pro-B-type Natriuretic Peptide; CK-MB, Creatine Kinase-MB; CK, Creatine Kinase; LDH, Lactate Dehydrogenase; AST, Aspartate Aminotransferase; ALT, Alanine Aminotransferase; BUN, Blood Urea Nitrogen; CRE, Creatinine; ALB, Albumin; Mg^2+^, Magnesium; Ca^2+^, Calcium; K^+^ − Potassium; P^−^, Inorganic Phosphate; FIB, Fibrinogen; D-D, D-dimer; hs-CRP, High-Sensitivity C-Reactive Protein.

### Univariable analysis of risk factors in mechanical complications in very elderly AMI patients

**Figure 2:**
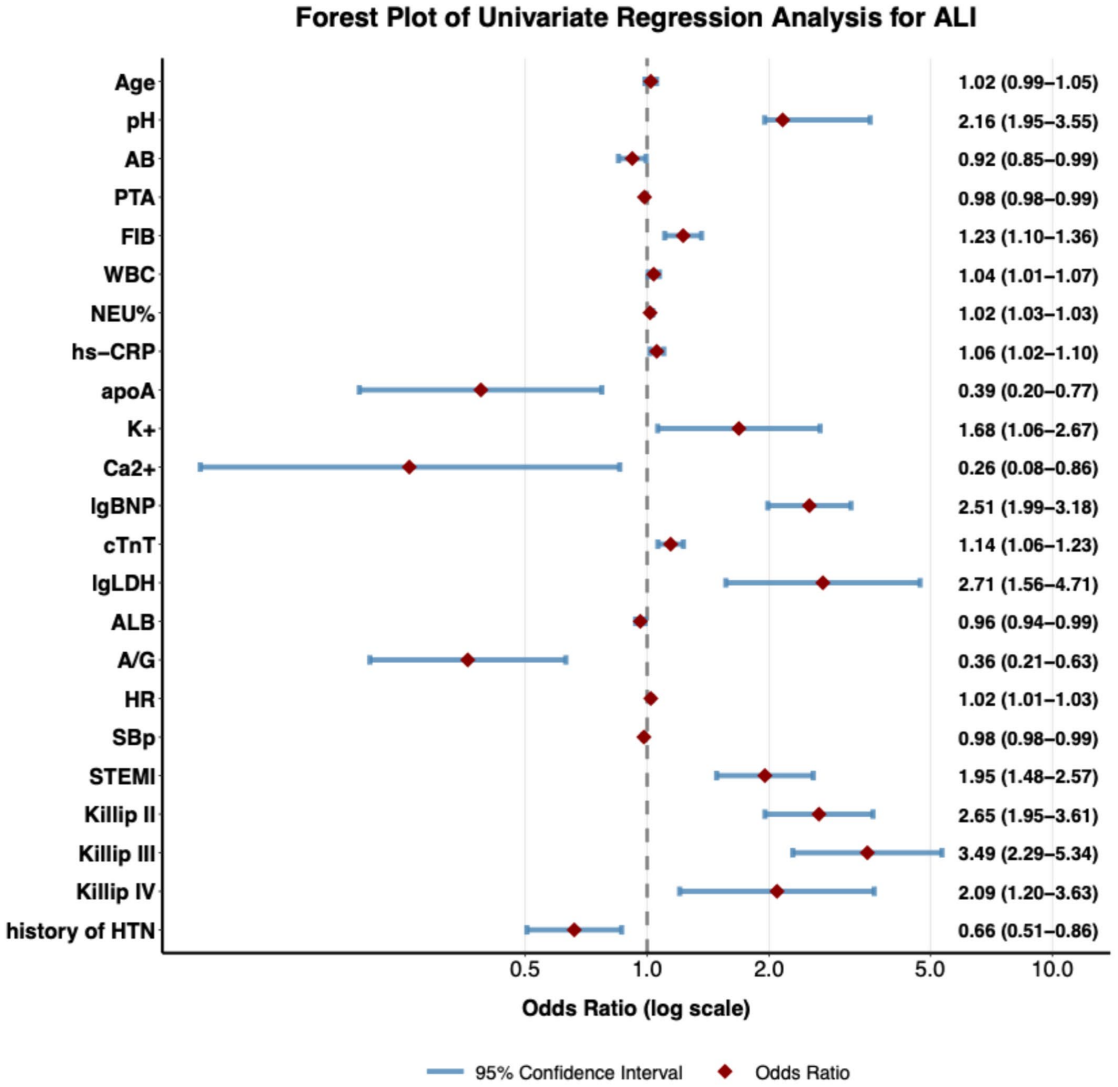
Forest plot of univariate predictors for mechanical complications in very elderly AMI patients. Forest plot of univariate logistic regression analysis, showing the strength of association between each clinical variable and the outcome event. Each dot in the plot represents the Odds Ratio (OR) of a variable, and the horizontal line indicates the 95% confidence interval. The diamond symbol represents the OR value, whose size is proportional to the effect size. The reference line (OR = 1) is indicated by a vertical dashed line; when the confidence interval crosses this line, it means that the variable has no statistically significant association with the outcome. The plot includes multiple types of variables such as age, laboratory indicators, vital signs, clinical diagnoses, and medical history, demonstrating their independent predictive value for the outcome event. All analyses were performed using a univariate logistic regression model, and the OR values along with their confidence intervals are shown to the right of each variable.

Univariate logistic regression identified multiple significant predictors for mechanical complications in very elderly AMI patients (Figure 2). STEMI patients are at a higher risk of developing mechanical complications (OR = 1.95, 95%CI 1.48–2.57). Markers of myocardial injury and stress demonstrated strong associations, with elevated cardiac troponin T (cTnT: OR = 1.14, 95% CI 1.06–2.67) and logarithmic N-terminal pro-B-type natriuretic peptide (lgBNP: OR = 2.51, 95% CI 1.99–3.18) exhibiting substantial effects. Inflammatory activation was consistently prognostic, as evidenced by significant associations with leukocytosis (WBC: OR = 1.04, 95% CI 1.01–1.07), elevated fibrinogen (FIB: OR = 1.23, 95% CI 1.11–1.36), and high-sensitivity C-reactive protein (hs-CRP: OR = 1.06, 95% CI 1.02–1.11). Hemodynamic compromise was reflected in Killip class gradation (Killip III: OR = 3.49, 95% CI 2.29–5.34; Killip IV: OR = 2.09, 95% CI 1.21–3.63). Metabolic derangements emerged as key predictors, including hypealbuminemia (ALB: OR = 0.96, 95% CI 0.94–0.99) and hyperkalemia (K^+^ = 1.6, 95% CI 1.06–2.67). Paradoxically, hypertension history (OR = 0.66, 95% CI 0.51–0.87) conferred reduced risk.

### Multivariable analysis of risk factors in mechanical complications in very elderly AMI patients

Multivariable logistic regression analyses identified independent predictors for mechanical complications across progressively adjusted models (Table 4). In Model 1 (adjusted for age, sex, hypertension history, and diabetes history), hypertension history demonstrated a significant protective association (OR = 0.67, 95% CI 0.51–0.88), while other variables were non-significant. Model 2 (incorporating inflammatory markers alongside hypertension history) revealed fibrinogen (FIB: OR = 1.21, 95% CI 1.09–1.35) as a significant positive predictor, with the protective effect of hypertension history persisting and strengthening (OR = 0.63, 95% CI 0.48–0.83). The fully adjusted Model 3 identified four independent predictors: higher Killip class III/IV (OR = 2.99, 95% CI 1.61–5.56), elevated neutrophil percentage (OR = 1.05, 95% CI 1.02–1.08), hyperkalemia (K^+^: OR = 1.70, 95% CI 1.08–2.70), and lower serum albumin (ALB: OR = 0.92, 95% CI 0.87–0.97). Hypertension history maintained its protective association (OR = 0.50, 95% CI 0.30–0.82), while age and sex remained non-significant.

**Table 4:** Multivariate regression analysis of mechanical complications.

Variable	Model 1		Model 2		Model 3	
	OR (95% CI)	*p* value	OR (95% CI)	*p* value	OR (95% CI)	*p* value
Age, year	1.02 (0.98, 1.05)	0.306	1.01 (0.98, 1.05)	0.425	1.03 (0.97, 1.10)	0.360
Male, *n* (%)	0.79 (0.60, 1.05)	0.099	0.85 (0.64, 1.13)	0.260	0.65 (0.39, 1.09)	0.102
HX of HTN	0.67 (0.51, 0.88)	0.004	0.63 (0.48, 0.83)	0.001	0.50 (0.30, 0.82)	0.006
HX of T2DM	0.89 (0.66, 1.20)	0.437	-		-	
FIB	-		1.21 (1.09, 1.35)	< 0.001	-	
NEU%	-		1.02 (1.00, 1.03)	0.037	1.05 (1.02, 1.08)	< 0.001
K^+^	-		-		1.70 (1.08, 2.70)	0.023
ALB	-		-		0.92 (0.87, 0.97)	0.002
Killip III/IV	-		-		2.99 (1.61, 5.56)	< 0.001

Model 1: adjusted for age, sex, history of hypertension, history of T2DM; Model 2: adjusted for history of hypertension, history of hypertension, FIB, NEU%; Model 3: adjusted for age, sex, history of hypertension, NEU%, K^+^, ALB, Killip III/IV.

The sensitivity analysis, comparing the final model derived from stepwise selection with a full model containing all pre-specified covariates, confirmed the robustness of our findings. The five key independent predictors identified in the final model exhibited highly consistent effect estimates and maintained their strong statistical significance (*p* < 0.05) in the full model. Furthermore, the final model demonstrated a superior balance of fit and parsimony, as objectively indicated by its lower Akaike (AIC) and Bayesian (BIC) Information Criterion values compared to the full model (Supplementary Table 5).

## Discussion

This large cohort study provides a comprehensive analysis of mechanical complications in a vulnerable population of very elderly patients (≥75 years) presenting with AMI. Our findings delineate distinct epidemiological patterns, clinical predictors, and pathophysiological profiles associated with mechanical complications, offering crucial insights for risk stratification and potential therapeutic targeting in this growing demographic.

The overall incidence of mechanical complications in our very elderly AMI cohort was 9.6%, which is consistent with existing research data, confirming their continued clinical relevance despite advances in reperfusion therapy (15). The exceptionally high prevalence of VA, constituting 92.8% of mechanical complications in our very elderly AMI cohort, underscores its clinical dominance over rupture complications or papillary muscle pathology (16). This predominance arises from interconnected factors specific to advanced age and management. The inherent vulnerability of the senescent heart, marked by increased stiffness and impaired repair, creates a substrate favoring chronic maladaptive remodeling over acute rupture (17–19). Critically, VA exhibited a strong association with large anterior infarcts (71.2% of cases), where proximal LAD occlusion causes extensive transmural necrosis in relatively thinner, aged myocardium particularly susceptible to progressive stretching under systolic pressure (20, 21). Furthermore, successful reperfusion, achieved in 81.7% of VA patients, presents a paradoxical effect: while salvaging myocardium overall, it often rescues a rim of ischemic but non-contractile border zone tissue in the aged heart (22, 23). This structurally compromised, akinetic rim stretches continuously, facilitating chronic aneurysm formation rather than contributing to function. This mechanism contrasts sharply with rupture complications (VSR/FWR), which demonstrated a predilection for non-reperfused infarcts (50.0 and 42.9%, respectively), where larger areas of homogeneous transmural necrosis create brittle zones highly vulnerable to catastrophic tearing during early inflammatory weakening. Thus, the convergence of aged myocardial vulnerability, anterior infarct location, and the specific remodeling effects of reperfusion collectively drive VA as the dominant mechanical complication trajectory in very elderly AMI patients.

Our analysis identified distinct demographic risk profiles for specific mechanical complications. Female sex was independently associated with a significantly higher risk of cardiac rupture, the most immediately lethal complication, highlighting a critical sex-based vulnerability that warrants heightened acute vigilance. Conversely, advancing age, particularly beyond 75 years, was powerfully correlated with a complication profile overwhelmingly dominated by left ventricular aneurysm. This shift from acute rupture to adverse remodeling in the elderly necessitates a parallel shift in clinical focus from solely preventing catastrophe to also managing the long-term sequelae of chronic left ventricular dysfunction. These findings collectively advocate for a refined, patient-tailored approach to post-AMI monitoring, where clinical suspicion is guided by a patient’s specific demographic risk profile.

This study identifies a characteristic complication profile in elderly AMI patients where structural remodeling and electrophysiological instability predominate. Left ventricular aneurysms serve dual roles as both manifestations of structural damage and arrhythmogenic substrates that exacerbate cardiac dysfunction through geometric and hemodynamic alterations. Age-related factors including myocardial fibrosis, microcirculatory impairment, and multimorbidity collectively drive this pattern. These findings support implementing stratified management that maintains acute mechanical complication vigilance while establishing systematic monitoring and secondary prevention protocols to address the more prevalent long-term risks of cardiac dysfunction and arrhythmias.

The observed concentration of mechanical complications—particularly VSR, FWR, and papillary muscle pathology—within very elderly AMI patients exhibiting impaired renal function (eGFR ≤45 mL/min/1.73m^2^) suggests a multifactorial pathophysiological interplay. Protein-bound uremic retention solutes (e.g., indoxyl sulfate, p-cresyl sulfate) accumulate in renal dysfunction, directly impairing extracellular matrix integrity via oxidative stress-mediated inhibition of collagen cross-linking and suppression of autophagy, thereby increasing myocardial wall fragility (24). Concurrently, a chronic microinflammatory state synergizes with acute post-infarction inflammation, creating a biphasic cytokine surge that disproportionately upregulates matrix metalloproteinase (MMP-9) activity relative to tissue inhibitors (TIMPs), accelerating degradation of the infarct border zone. Hemodynamically, sodium retention and neurohormonal activation (renin-angiotensin-aldosterone system [RAAS] and sympathetic overdrive) elevate ventricular wall stress through increased preload and afterload, potentiating infarct expansion (24, 25). Microvascular dysfunction, characterized by endothelial nitric oxide synthase (eNOS) downregulation and impaired capillary density, further extends transmural necrosis, creating homogeneous zones vulnerable to shear forces. Paradoxically, VA predominance in preserved eGFR patients (91.3% of VA cases) likely reflects distinct mechanisms: successful reperfusion in this subgroup salvages a rim of ischemic but non-contractile myocardium. In aged hearts with limited regenerative capacity, this akinetic tissue undergoes maladaptive stretching under systolic pressure, facilitated by acute hypophosphatemia-induced ATP depletion impairing contractile recovery (26). Thus, while renal impairment promotes rupture through toxin-mediated matrix fragility, inflammation-protease imbalance, and hemodynamic stress, preserved renal function—coupled with higher reperfusion rates—predisposes to VA by enabling border zone salvage without functional recovery, highlighting a critical divergence in complication pathways dictated by renal-metabolic-inflammatory crosstalk.

Patients developing mechanical complications exhibited a distinct clinical profile compared to those without complications. While demographic factors (age, sex) were comparable, the MC group had a significantly lower prevalence of hypertension history (53.0% vs. 63.0%, *p* = 0.002). This counterintuitive protective association of hypertension history persisted as a significant independent predictor in multivariable analyses, warranting further investigation. Potential explanations include altered myocardial remodeling due to chronic pressure overload, differences in medication adherence (e.g., ACEi/ARBs, beta-blockers), or intrinsic biological factors associated with both hypertension development and resilience to mechanical failure.

Clinically, the MC group presented with greater hemodynamic instability, evidenced by significantly lower systolic blood pressure (115.90 vs. 123.45 mmHg, *p* = 0.001). They also demonstrated greater clinical severity at presentation: STEMI was markedly more frequent (60.6% vs. 44.1%, *p* < 0.001), and patients were significantly more likely to present with advanced Killip class (Killip III-IV: 22.5% vs. 13.3%, *p* < 0.001). This association of higher Killip class (III/IV) with mechanical complications was confirmed as a powerful independent predictor in the fully adjusted multivariable model (OR = 2.99). These findings collectively paint a picture of patients experiencing larger, more transmural infarctions leading to both profound hemodynamic compromise and an increased mechanical burden on the infarcted myocardium, predisposing to complications.

The observation that a history of hypertension conferred a protective effect against Mechanical complications in very elderly AMI patients—an association that persisted after multivariable adjustment for age, sex, comorbidities, and renal function—represents a notable and counterintuitive finding. However, the interpretation of this paradoxical relationship requires caution and must acknowledge the potential for unmeasured confounding and the speculative nature of the underlying mechanisms. For instance, the lack of detailed data on pre-admission antihypertensive medication adherence and dosages precludes definitive conclusions, as the observed protective effect could be largely attributable to the widespread use of cardioprotective agents like RAAS inhibitors and beta-blockers in this population.

Nevertheless, several biologically plausible, though unproven, hypotheses may be posited to explain this association. Chronic hypertension often induces left ventricular hypertrophy (LVH), characterized by cardiomyocyte enlargement and increased collagen deposition (22). While maladaptive in the long term, this structural remodeling in the geriatric heart might theoretically fortify the myocardial architecture against acute tensile failure during infarction. The thickened ventricular wall could reduce systolic wall stress, potentially attenuating distending forces on the necrotic border zone. Furthermore, long-standing hypertension might prime compensatory neurohormonal pathways, including a preconditioning-like upregulation of heat shock proteins and enhanced myocardial antioxidant capacity, which could mitigate reperfusion injury and oxidative damage to extracellular matrix proteins. Additionally, hypertension-driven coronary collateralization might limit infarct transmurality—a key prerequisite for Mechanical complications—by maintaining microperfusion in ischemic territories.

Therefore, while the exact mechanisms remain uncertain and may be confounded by medication effects, the aggregate effect of structural fortification, molecular preconditioning, and cardioprotective pharmacotherapy *might* collectively contribute to a net protective phenotype against catastrophic myocardial wall failure in this specific aged demographic. This hypothesis-generating observation warrants further investigation in prospective studies designed to meticulously control for potential confounding factors; in subsequent analyses, we will strive to collect detailed data on patients’ medication history to quantify the specific impact of drug confounding on this association. Should the association be confirmed in future studies, it may imply that in some elderly patients with chronic hypertension, the myocardium exhibits a unique biological adaptation to the stress response of acute infarction—a finding that provides a valuable direction for research into potential protective mechanisms.

The pronounced biomarker differences offer valuable insights into the underlying pathophysiology driving Mechanical complications. The MC group exhibited robust evidence of systemic and local inflammation: significantly elevated WBC, NEU%, and hs-CRP. Crucially, NEU% emerged as a strong independent predictor in the final multivariable model (OR = 1.05). Neutrophils are key mediators of early tissue injury post-MI, releasing proteases and reactive oxygen species that directly contribute to myocardial matrix degradation and weakening, potentially facilitating both rupture and aneurysm formation.

Markers of myocardial injury and stress were substantially elevated in the MC group, including significantly cTnT and markedly elevated NT-proBNP. While cTnT was a strong univariate predictor, its effect was attenuated in multivariable models, suggesting its elevation reflects infarct size but may not be an independent driver of Mechanical complications beyond the associated inflammation and hemodynamics. The extreme elevation of NT-proBNP underscores the severe ventricular wall stress and dysfunction inherent in these complications.

A prothrombotic state was evident in the MC group, characterized by elevated FIB and D-dimer. FIB was a significant predictor in the model incorporating inflammatory markers. Metabolic derangements were also prominent: hypocalcemia, hyperkalemia, hypoalbuminemia, and lower LDL-C. Hypealbuminemia and hyperkalemia were identified as independent predictors in the final model (OR = 0.92 and OR = 1.70, respectively). Hypoalbuminemia may reflect malnutrition-inflammation complex syndrome, impairing tissue repair, while hyperkalemia could indicate more severe cellular breakdown or renal handling issues, potentially exacerbating electrical instability in vulnerable myocardium. Lower serum calcium might impair myocardial contractility and hemostasis. Lower PCO_2_ and higher pH suggest compensatory hyperventilation in response to severe hemodynamic compromise or acidosis.

The multivariable analysis progressively refined our understanding of independent risk factors. Hypertension history consistently demonstrated a protective association, persisting and strengthening in more adjusted models (final OR = 0.50). The final model (Model 3) identified four robust independent predictors alongside hypertension history: Killip class III/IV (OR = 2.99), elevated NEU% (OR = 1.05), Hyperkalemia (K^+^) (OR = 1.70), and higher serum Albumin (ALB) (OR = 0.92). This combination highlights the convergence of clinical severity (Killip class), acute inflammatory response, metabolic derangement, and nutritional/inflammatory status (ALB) in determining MC risk. The prominence of NEU% and ALB suggests readily available admission laboratory values, combined with Killip class assessment, could form the basis for practical bedside risk stratification tools for Mechanical complications in very elderly AMI patients. Based on multivariate regression analysis, this study developed a risk prediction model incorporating four admission-available indicators: Killip class III/IV, elevated neutrophil percentage, hyperkalemia, and hypoalbuminemia. This model provides a practical tool for risk stratification of mechanical complications in elderly patients with acute myocardial infarction (AMI), and helps guide targeted clinical interventions: enhancing monitoring of cardiac function and myocardial structure in high-risk patients, promptly correcting inflammatory and metabolic disorders, and optimizing the allocation of critical care resources. This strategy translates epidemiological findings into a clinical practice tool, which is expected to improve the clinical management of such high-risk patients.

Several limitations of this study should be acknowledged. First, as an observational study, the associations identified are subject to potential residual confounding. Although we adjusted for numerous clinical variables, unmeasured factors such as pre-morbid functional status, the extent of coronary collateralization, detailed medication adherence, and specific echocardiographic parameters of left ventricular remodeling may have influenced the results. The paradoxical protective effect of a hypertension history, for instance, could be partially explained by such unmeasured confounders. Second, the absence of systematic data on the time from symptom onset to reperfusion and the lack of core laboratory-adjudicated angiographic or cardiac magnetic resonance imaging data preclude a more granular analysis of the relationships between ischemic time, myocardial salvage, and the risk of specific Mechanical complications. Finally, this was a single-center study, which may limit the generalizability of our predictive model to other populations.

What’s more, the overwhelming predominance of ventricular aneurysm within our MC cohort means that the numbers of other mechanical complications (e.g., free-wall rupture, ventricular septal rupture) were very small. This precluded statistically robust multivariate analyses for these specific subtypes, and their characterization in this study should be interpreted as descriptive and hypothesis-generating.”

## Conclusion

In summary, ventricular aneurysm represents the predominant mechanical complication in very elderly AMI patients, particularly associated with anterior infarct location and reperfusion therapy. Patients developing these complications typically present with STEMI, hemodynamic compromise, and distinct biomarker profiles reflecting intense inflammation (notably neutrophilia), myocardial injury, and metabolic disturbances. Our analysis identifies Killip class III/IV, elevated neutrophil percentage, hyperkalemia, and hypoalbuminemia as independent predictors, while revealing a paradoxical protective association with hypertension history. These findings collectively propose a clinically actionable risk stratification framework; however, this model requires prospective validation in external cohorts to confirm its generalizability and potential for guiding targeted interventions in this vulnerable population. Future studies should focus on three key areas: first, validating this risk model in prospective, multicenter cohorts; second, exploring the feasibility of integrating indicators such as neutrophil percentage, serum potassium, and albumin into existing risk scores (e.g., GRACE); and third, identifying preventive intervention measures for high-risk patients.

## Data Availability

The original contributions presented in the study are included in the article/supplementary material, further inquiries can be directed to the corresponding author.
