# Supplementary material for: Risk factors for mechanical complications in very elderly patients with acute myocardial infarction
Source: Front Med (Lausanne). 2025 Dec 2;12:1714080. doi: 10.3389/fmed.2025.1714080 (PMC12705586; doi:10.3389/fmed.2025.1714080)
Supplement: Supplementary file 1 [file Table_1.docx]

### **Supplement Table 1. Impact of Exclusion Criteria on the Reported Incidence of Mechanical Complications in the Acute Myocardial Infarction (AMI) Cohort**

| **Cohort Description** | **Total Patient Number (n)** | **Patients with Mechanical Complications (n)** | **Incidence of Mechanical Complications (%)** |
| --- | --- | --- | --- |
| **Total AMI Population Screened** | 2490 | 259 | 10.4% |
| **Excluded: Pre-admission Mechanical Complications** | 23 | 23 | 100.0% |
| **Final In-Hospital Study Cohort** | 2467 | 236 | 9.6% |

**Note**: This table clarifies that while the primary analysis focused on the 9.6% in-hospital incidence, the overall burden of AMI-related mechanical complications, including those present on admission, was significantly higher at 10.4%.
