# Supplementary material for: Risk factors for mechanical complications in very elderly patients with acute myocardial infarction
Source: Front Med (Lausanne). 2025 Dec 2;12:1714080. doi: 10.3389/fmed.2025.1714080 (PMC12705586; doi:10.3389/fmed.2025.1714080)
Supplement: Supplementary file 2 [file Table_2.docx]

**Supplement Table 2. Sex-Based Differences in the Incidence of Mechanical Complications**

| **Variables** | **Male (n=1,621)** | **Female (n=846)** | **P-value** |
| --- | --- | --- | --- |
| **Overall Incidence** | 144 (8.9%) | 92 (10.9%) | 0.085 |
| **Type of Complication** |  |  |  |
| Cardiac Rupture | 2 (0.12%) | 5 (0.59%) | **0.024** |
| Ventricular Septal Rupture | 4 (0.25%) | 4 (0.47%) | 0.288 |
| Papillary Muscle Rupture | 5 (0.31%) | 3 (0.35%) | 0.560 |
| Left Ventricular Aneurysm | 136 (8.4%) | 83 (9.8%) | 0.191 |
