# Supplementary material for: Risk factors for mechanical complications in very elderly patients with acute myocardial infarction
Source: Front Med (Lausanne). 2025 Dec 2;12:1714080. doi: 10.3389/fmed.2025.1714080 (PMC12705586; doi:10.3389/fmed.2025.1714080)
Supplement: Supplementary file 3 [file Table_3.docx]

**Supplement Table 3: Incidence and Profile of Mechanical Complications by Age Group**

| **Variables** | **<65 Years (n=10,391)** | **65-75 Years (n=4,710)** | **>75 Years (n=2,467)** |
| --- | --- | --- | --- |
| **Overall Incidence** | 713 (6.86%) | 408 (8.66%) | 236 (9.57%) |
| **Complication Profile (n, % within group)** | (n=713) | (n=408) | (n=236) |
| Left Ventricular Aneurysm | 637 (89.3%) | 354 (86.8%) | 213 (90.3%) |
| Ventricular Septal Rupture | 37 (5.2%) | 26 (6.4%) | 8 (3.4%) |
| Papillary Muscle Rupture | 19 (2.7%) | 15 (3.7%) | 8 (3.4%) |
| Cardiac Rupture (FWR) | 20 (2.8%) | 13 (3.2%) | 7 (3.0%) |
