# Supplementary material for: Risk factors for mechanical complications in very elderly patients with acute myocardial infarction
Source: Front Med (Lausanne). 2025 Dec 2;12:1714080. doi: 10.3389/fmed.2025.1714080 (PMC12705586; doi:10.3389/fmed.2025.1714080)
Supplement: Supplementary file 4 [file Table_4.docx]

**Supplement Table 4: Comprehensive Spectrum of Complications in the Elderly AMI Cohort**

| **Complication Category** | **Specific Complication** | **Number of Cases** | **Incidence (%)** |
| --- | --- | --- | --- |
| **Major Structural Complications** | **All Major Structural Complications** | **236** | **9.57** |
|  | Left Ventricular Aneurysm | 213 | 8.63 |
|  | Ventricular Septal Rupture | 8 | 0.32 |
|  | Papillary Muscle Rupture | 8 | 0.32 |
|  | Cardiac Free Wall Rupture | 7 | 0.28 |
| **Ventricular Function & Thrombus** | **Any Significant Ventricular Dysfunction/Thrombus** | **395** | **16.01** |
|  | Significant LV Dysfunction (LVEF <35%) | 296 | 12.00 |
|  | Significant RV Dysfunction | 123 | 4.99 |
|  | Left Ventricular Thrombus | 25 | 1.01 |
| **Significant Dysrhythmias** | **Any Significant Dysrhythmia** | **395** | **16.01** |
|  | New-Onset Atrial Fibrillation/Flutter | 222 | 9.00 |
|  | High-Grade Atrioventricular Block | 99 | 4.01 |
|  | Sustained Ventricular Tachycardia/Ventricular Fibrillation | 99 | 4.01 |

Abbreviation: LVEF: Left Ventricular Ejection Fraction. Some patients experienced multiple complication hence the sum of individual complications may exceed the total for the category.
