# Supplementary material for: Risk factors for mechanical complications in very elderly patients with acute myocardial infarction
Source: Front Med (Lausanne). 2025 Dec 2;12:1714080. doi: 10.3389/fmed.2025.1714080 (PMC12705586; doi:10.3389/fmed.2025.1714080)
Supplement: Supplementary file 5 [file Table_5.docx]

**Supplement Table 5. Sensitivity Analysis: Comparison of Multivariable Models for Predictors of Mechanical Complications**

| Variable | Full Model | | Final Model | |
| --- | --- | --- | --- | --- |
|  | Adjusted OR (95% CI) | P Value | Adjusted OR (95% CI) | P Value |
| **Hypertension History** | 0.54 (0.40 - 0.73) | < 0.001 | 0.50 (0.37 - 0.67) | < 0.001 |
| **Killip Class III/IV** | 2.85 (2.02 - 4.02) | < 0.001 | 2.99 (2.15 - 4.16) | < 0.001 |
| **Neutrophil Percentage (%)** | 1.05 (1.03 - 1.07) | < 0.001 | 1.05 (1.03 - 1.07) | < 0.001 |
| **Serum Potassium (mmol/L)** | 1.65 (1.35 - 2.02) | < 0.001 | 1.70 (1.40 - 2.06) | < 0.001 |
| **Serum Albumin (g/L)** | 0.93 (0.90 - 0.96) | < 0.001 | 0.92 (0.89 - 0.95) | < 0.001 |
| Age | 1.02 (0.98 - 1.05) | 0.362 | - | - |
| Male Sex | 0.85 (0.63 - 1.14) | 0.278 | - | - |
| Diabetes Mellitus History | 1.10 (0.82 - 1.47) | 0.532 | - | - |

| **Model Statistics** | **Full Model** | **Final Model** |
| --- | --- | --- |
| Number of Observations | 2467 | 2467 |
| Number of Events (Any Mechanical complications) | 236 | 236 |
| Akaike Information Criterion (AIC) | 1621.5 | 1615.8 |
| Bayesian Information Criterion (BIC) | 1674.2 | 1643.9 |

**Abbreviations:** OR, Odds Ratio; CI, Confidence Interval.

**Note:** The Full Model included all pre-specified covariates of clinical interest. The Final Model was derived using stepwise backward elimination with a retention threshold of *P*<0.05. The model was fitted on the entire cohort of 2467 patients, among which 236 experienced the composite endpoint of any mechanical complication.
