# Supplementary material for: Risk factors for mechanical complications in very elderly patients with acute myocardial infarction
Source: Front Med (Lausanne). 2025 Dec 2;12:1714080. doi: 10.3389/fmed.2025.1714080 (PMC12705586; doi:10.3389/fmed.2025.1714080)
Supplement: Supplementary file 6 [file Table_6.docx]

**Supplement Table 6: Utilization of Mechanical Circulatory Support Devices in the Study Cohort**

| **MCS Device** | **Total Patients Requiring Support (n)** | **Utilization Rate (%)** | **Patients with Mechanical Complications (n=236)** | **Patients without Mechanical Complications (n=2231)** |
| --- | --- | --- | --- | --- |
| Any MCS | 321 | 13.0% | 98 (41.5%) | 223 (10.0%) |
| IABP | 247 | 10.0% | 71 (30.1%) | 176 (7.9%) |
| VA-ECMO | 49 | 2.0% | 22 (9.3%) | 27 (1.2%) |
| LVAD | 38 | 1.5% | 15 (6.4%) | 23 (1.0%) |

Abbreviations: IABP, intra-aortic balloon pump; VA-ECMO, veno-arterial extracorporeal membrane oxygenation; MCS, mechanical circulatory support, LVAD: left ventricular assist device
