# Supplementary material for: Risk factors for mechanical complications in very elderly patients with acute myocardial infarction
Source: Front Med (Lausanne). 2025 Dec 2;12:1714080. doi: 10.3389/fmed.2025.1714080 (PMC12705586; doi:10.3389/fmed.2025.1714080)
Supplement: Supplementary file 7 [file Table_7.docx]

**Supplement Table 7: In-Hospital Medication Profile of the Study Cohort**

| Medication | Total Cohort (n=2467) | Patients with Mechanical Complications (n=236) | Patients without Mechanical Complications (n=2231) |
| --- | --- | --- | --- |
| **Dual Antiplatelet Therapy (DAPT)** | 2,321 (94.1%) | 218 (92.4%) | 2103 (94.3%) |
| Aspirin + Clopidogrel | 1729 (70.1%) | 172 (72.9%) | 1557 (69.8%) |
| Aspirin + Ticagrelor | 592 (24.0%) | 46 (19.5%) | 546 (24.5%) |
| **Beta-Blockers** | 1926 (78.1%) | 168 (71.2%) | 1758 (78.8%) |
| Metoprolol | 1753 (71.1%) | 152 (64.4%) | 1601 (71.8%) |
| **Renin-Angiotensin System Inhibitors** | 1728 (70.0%) | 142 (60.2%) | 1586 (71.1%) |
| ACEI/ARB | 1553 (63.0%) | 131 (55.5%) | 1,22 (63.7%) |
| ARNI (Sacubitril/Valsartan) | 175 (7.1%) | 11 (4.7%) | 164 (7.4%) |
| **SGLT2 Inhibitors** | 445 (18.0%) | 28 (11.9%) | 417 (18.7%) |
| Dapagliflozin | 445 (18.0%) | 28 (11.9%) | 417 (18.7%) |
| **Diuretics** | 889 (36.0%) | 127 (53.8%) | 762 (34.2%) |
| **Anticoagulants** | 642 (26.0%) | 89 (37.7%) | 553 (24.8%) |
