# Supplementary material for: Risk factors for mechanical complications in very elderly patients with acute myocardial infarction
Source: Front Med (Lausanne). 2025 Dec 2;12:1714080. doi: 10.3389/fmed.2025.1714080 (PMC12705586; doi:10.3389/fmed.2025.1714080)
Supplement: Supplementary file 8 [file Table_8.docx]

**Supplement Table 8: Patient Admission Pathways and Thrombolysis Utilization**

| Admission Pathway | Number of Patients | Percentage of Total Cohort | Patients Receiving Thrombolysis |
| --- | --- | --- | --- |
| Direct Admission from Field | 1,849 | 75.0% | 0 (0%) |
| Transfer from Other Facility | 618 | 25.0% | 52 (8.4%) |
| Total Cohort | 2,467 | 100% | 52 (2.1% of total) |
